# An exploratory analysis of adult daily smokers’ experiences using e-cigarettes in smoke-free places

**DOI:** 10.18332/tid/98958

**Published:** 2018-11-19

**Authors:** Sabrina L. Smiley, Elexis C. Kierstead, Emily Harvey, Haneen Abudayyeh, Jennifer L. Pearson

**Affiliations:** 1 Department of Preventive Medicine, Institute for Health Promotion and Disease Prevention Research, Keck School of Medicine, University of Southern California, Los Angeles, United States; 2 Schroeder Institute for Tobacco Research and Policy Studies at Truth Initiative, Washington, United States; 3 Milken Institute School of Public Health, George Washington University, Washington, United States; 4 School of Community Health Sciences, University of Nevada, Reno, United States

**Keywords:** qualitative study, e-cigarettes, cigarette smokers, smoke-free environments

## Abstract

**INTRODUCTION:**

Evidence indicates that one reason cigarette smokers value e-cigarettes is the ability to use them in places where smoking is not permitted. We sought to: 1) explore adult daily smokers’ experiences using e-cigarettes in the context of smoke-free places; and 2) describe smokers’ perceptions of bystanders’ reactions.

**METHODS:**

Twenty adult daily smokers in Washington, DC initiated e-cigarettes for three weeks and completed in semi-structured interviews at the end of each week. All interviews (n=60) were digitally-recorded, transcribed verbatim, imported into NVivo 10.0, and analyzed using thematic analysis methodology.

**RESULTS:**

The sample had a mean age of 37.9 years and 18 participants reported having smoked their first cigarette by age 18. Common themes included descriptions of: 1) uncertainty about whether smoke-free policies included e-cigarettes; 2) using e-cigarettes in smoke-free places (e.g. restaurants, workplace, public transit-bus and rail); 3) approaches to e-cigarette use in smoke-free places as part of a complex decision-making process, ranging from testing and establishing the social and spatial boundaries of e-cigarette use, to confining e-cigarette use to inside their home; and 4) favorable, unfavorable, and impartial reactions from bystanders facilitated or impeded e-cigarette use, indicating social approval/social disapproval.

**CONCLUSIONS:**

Results suggest a continuum of factors, including smoke-free policies and reactions from bystanders may facilitate or impede e-cigarette use among smokers in environments where a smoke-free imperative is well-established. As e-cigarette use evolves, study findings indicate the importance of the social environment and how it could affect those switching from cigarettes to e-cigarettes.

## INTRODUCTION

In the United States, electronic cigarette (e-cigarette, e-cig) use is most common among adult (≥18 years) cigarette smokers^1,2^. Empirical evidence indicates that smokers perceive e-cigarettes as less harmful than cigarettes, as cessation aids, and as alternative products that can be used in ‘no smoking’ places/situations^3-6^. Past studies^3,5,7^ have found that smokers’ perceptions of e-cigarettes have been largely influenced by tobacco industry marketing strategies that promote e-cigarettes as smokeless, odorless, and discreet products that can be used anywhere, anytime.

We currently know little about e-cigarette use among adult daily smokers in the context of smoke-free environments. A cross-sectional study of national data by Shi et al.^5^ found that current adult smokers reported using e-cigarettes in smoke-free places like bars, restaurants, and places of employment. A recent qualitative study^8^ found that e-cigarettes were used by dual users (i.e. cigarette smokers and e-cigarette users) in places or situations when cigarette smoking was either not allowed (e.g. public transit, shopping mall) or socially disapproved by family (e.g. home/indoors). This suggests that smokers may choose to use e-cigarettes in places/situations where they cannot smoke cigarettes, and may perceive e-cigarette use as convenient, more socially acceptable than smoking cigarettes, and lawful.

To date, research investigating the role of social approval/disapproval of e-cigarette use in smoke-free environments is sparse. One study^9^ reported that e-cigarette use in restaurants and workplaces is viewed as more socially acceptable than cigarettes. Similarly, evidence^10-12^ indicates that public support for policies to restrict e-cigarette use in public places is considerably lower than for smoking bans. This suggests that e-cigarette use in smoke-free environments may be socially acceptable, which may facilitate use among smokers initiating e-cigarettes. Nevertheless, to our knowledge, no study has investigated how adult daily smokers understand the role of smoking restrictions in e-cigarette use, and how social norms surrounding cigarettes and e-cigarettes affect product use in smoke-free places. Therefore, it is important to identify factors that explain why adult daily smokers may or may not use e-cigarettes in smoke-free places/situations.

Through one-on-one interviews with adult daily smokers in Washington, District of Columbia (DC), we sought to: 1) explore adult daily smokers’ experiences using e-cigarettes in the context of smoke-free places/situations; and 2) describe smokers’ perceptions of bystanders’ reactions to their e-cigarette use in smoke-free places/situations.

## METHODS

### Social setting

Washington, DC is an informative setting in which to investigate the use of e-cigarettes in smoke-free environments. In April 2006, the DC City Council enacted a 100% smoke-free indoor air policy, and in January 2007 the policy was implemented in restaurants, bars, and nightclubs^13^. In November 2016, the DC City Council extended smoke-free indoor air policy to cover e-cigarette use, including use in the workplace, public transit, and sporting events^14^. It is important to note that while data were being collected for the current study, e-cigarettes had not yet been included in the DC smoke-free policy.

### Sample and recruitment

Data were drawn from the parent study (‘Moment Study’), a three-week intensive longitudinal mixed-methods study. The Moment Study investigated e-cigarette initiation and cigarette displacement among adult daily smokers in Washington, DC, and is described in detail elsewhere^15^. Briefly, the Moment Study’s design featured concurrent collection of multiple data streams, including: 1) ecological momentary assessment, 2) geotracking, 3) three semi-structured interviews, and 4) biosamples. To elicit participants’ experiences and their views on using e-cigarettes in smoking restricted areas, only data from the semi-structured interviews are included in this paper, as they focus on the subjective perspective of the observed participants.

Adult daily smokers interested in using e-cigarettes were recruited via public online postings (e.g. Craigslist), paid advertisements (e.g. Washington Post Express), and flyers. Inclusion criteria included: 1) be 18 years or older, 2) reside in the Washington, DC metropolitan area, 3) report daily smoking of at least eight cigarettes a day for the past five years, 4) report no e-cigarette use in the past 30-days, and 5) not currently taking a smoking cessation medication (e.g. varenicline) or nicotine replacement therapy. Once enrolled, in-person procedures consisted of four office visits. All participants provided written informed consent and were compensated with up to $285 if they completed all study activities, including the three interviews. The Chesapeake Institutional Review Board approved all study procedures. For this qualitative analysis of initial understanding of e-cigarette use in smoke-free places/situations, three repeated-measures interviews (n=60) from 20 smokers in our sample of 107 were selected to be part of a subsample. This sample size reflects the ‘15 ± 10’ metrics for qualitative interview studies^16^.

### Study materials

Following the first office visit (baseline interview), two packs of five disposable NJOY King ‘cigalike’ e-cigarettes (3.0% nicotine) were provided to participants at the end of the interviews conducted at the second and third office visits. Participants were provided regular or menthol ‘cigalike’ e-cigarettes, depending on their cigarette brand flavor preference. At the second office visit, participants were instructed to try a minimum of three e-cigarette puffs per day over the course of the week. At the end of the third office visit, participants received two additional five-packs and were instructed to use them as they desired (including not at all) over the course of the week.

### Interview instruments

Three semi-structured interview guides^17^ were developed [by authors S.L.S. and J.L.P.], and included questions designed to examine how individuals understand the role of smoking restrictions on their e-cigarette use, and how social norms surrounding cigarettes and e-cigarettes affect product use (Table 1). The interview format allowed participants to respond extemporaneously to questions and discuss relevant topics at the second, third, and fourth office visits. The interview guide at the second office visit, focused on establishing rapport between the interviewer and participant, along with examining participants’ perceptions and utility of cigarettes in their lives, and perceptions of e-cigarettes. Sample questions included: ‘Tell me about yourself?’ and ‘What have you heard about e-cigarettes?’. As mentioned previously, two packs of five disposable NJOY King ‘cigalike’ e-cigarettes (3.0% nicotine) were first provided to participants at the second office visit, and again at the third office visit. The interview guide at the third and fourth office visits examined utility of e-cigarettes in their lives, including where they used e-cigarettes, others’ reactions, and perceived social norms. Sample questions included: ‘Can you tell me about the first time you tried an e-cigarette?’; ‘Where were you when you first tried an e-cigarette?’; ‘And how did it [using e-cigarettes] go the rest of the week?’. Probes included: ‘Describe the setting.’; ‘Were you inside or outside?’; ‘Were you alone or did you use them around others?’; and ‘How did others react?’.

**Table 1:** The Moment Study One-On-One Interview Assessment Methods

*Method*	*Description*	*Duration*	*Items/Instructions*
**Interview I**	Conducted at the second office visit and focused on establishing rapport, in addition to investigating participants’ perceptions and utility of cigarettes, and their perceptions of e-cigarettes. Sample questions included: “Tell me about yourself?” and “What have you heard about e-cigarettes?”	**30 minutes**	Participants were provided a week’s supply of e-cigarettes (2 NJOY King 5-packs; 3.0% nicotine) and were asked to try to take at least three puffs daily over the course of the week
**Interview II**	Conducted at the third office visit and investigated participants’ experience using e-cigarettes, including the role of social context, others’ reactions, and perceived social norms surrounding e-ciarette use and how these social norms influenced their own use	**30 minutes**	Participants’ e-cigarette supply was replenished (2 additional NJOY King 5-packs; 3.0% nicotine) and participants were instructed to use them as they desired (including not at all) over the course of the week
**Interview III**	Conducted at the fourth office visit and investigated participants’ different experiences with e-cigarettes compared to the week before, including others’ reactions, perceived social norms surrounding e-cigarette use, and how these social norms influenced their own use	**30 minutes**	**None**

### Data collection and analysis

Two of the authors (S.L.S. and E.H.) were trained in qualitative interviewing techniques and conducted interviews separately at participants’ second, third, and fourth office visits. Each interview lasted up to 30 minutes and took place in a dedicated interview room at Truth Initiative’s Washington, DC office. The interviews (n=60) were digitally recorded with consent, professionally transcribed verbatim, edited to remove identifiers, cross-checked for accuracy, and imported into NVivo 10.0, a qualitative software package that aids in the organization, coding, and analysis of qualitative data^18^. Guided by thematic analysis methodology^19^, four of the authors (S.L.S., E.C.K., E.H., H.A.) independently read the transcripts and held debriefing meetings that involved writing preliminary analytical interpretations of the data, and developing *a priori* codes (e.g. initial e-cig use, bystander reaction) and emergent codes (e.g. militant smoker, smoke e-cig) in relation to the interview guide questions, transcripts, and research questions. Two of the authors (S.L.S. and E.C.K.) independently coded a subset of transcripts line-by-line and compared them, which led to additional codes. The codebook consisted of each code, its complete definition, and an example of a quotation from a participant’s transcript. These authors (S.L.S. and E.C.K.) independently coded the larger set of transcripts, and themes were generated iteratively during review of coded transcripts. After multiple rounds of coding, the first and second authors (S.L.S. and E.C.K.) determined that coding saturation had been reached^20^, as new themes ceased to emerge. These authors also wrote analytical memos throughout the coding process and used them to confirm coding saturation. Lastly, the first author (S.L.S.) adjusted and re-checked the resulting themes against the transcripts^21^. All quotes are provided verbatim.

## RESULTS

### Participant characteristics

The sample (Table 2) comprised 10 women and 10 men (mean age M=37.9 years). Among participants, 10 self-identified as non-Hispanic Black and 10 self-identified as non-Hispanic White. Eighteen participants reported having smoked their first cigarette by the age of 18 years; 12 reported smoking menthol cigarettes; and 10 reported thinking about quitting in the next six months. Ten participants reported some college education; six reported college degrees; and four reported a high school degree as their highest level of educational attainment. Key themes are highlighted in Table 3.

**Table 2:** Characteristics of adult daily cigarette smokers (n=20 ), Washington, DC, 2015

*No.*	*Sex*	*Race/Ethnicity*	*Age*	*Highest education level*	*ASI*	*Cigarette type*	*Quit timeline*
1	F	non-Hispanic Black	55	Bachelors degree	16	Menthol	Thinking about quitting in the next 30 days
2	F	non-Hispanic Black	28	Some college	18	Menthol	Thinking about quitting in the next 6 months
3	F	non-Hispanic Black	42	Some college	20	Menthol	Not thinking about quitting
4	F	non-Hispanic Black	61	High school graduate	22	Menthol	Thinking about quitting in the next 30 days
5	M	non-Hispanic Black	61	Bachelors degree	15	Menthol	Thinking about quitting in the next 6 months
6	M	non-Hispanic Black	58	Some college	16	Menthol	Thinking about quitting in the next 30 days
7	M	non-Hispanic Black	24	High school graduate	16	Menthol	Thinking about quitting in the next 6 months
8	F	non-Hispanic White	32	Some college	13	Non-menthol	Thinking about quitting in the next 6 months
9	M	non-Hispanic White	29	Some college	15	Non-menthol	Thinking about quitting in the next 30 days
10	M	non-Hispanic White	26	Bachelors degree	16	Non-menthol	Thinking about quitting in the next 6 months
11	M	non-Hispanic White	29	Some college	16	Menthol	Thinking about quitting in the next 6 months
12	F	non-Hispanic Black	37	Some college	18	Menthol	Thinking about quitting in the next 30 days
13	F	non-Hispanic White	46	Some college	14	Non-menthol	Thinking about quitting in the next 6 months
14	F	non-Hispanic White	29	Some college	17	Menthol	Thinking about quitting in the next 6 months
15	M	non-Hispanic Black	56	High school graduate	18	Menthol	Thinking about quitting in the next 30 days
16	F	non-Hispanic White	43	Bachelors degree	15	Non-menthol	Thinking about quitting in the next 6 months
17	M	non-Hispanic White	32	Bachelors degree	17	Non-menthol	Thinking about quitting in the next 6 months
18	M	non-Hispanic Black	18	High school graduate	11	Menthol	Not thinking about quitting
19	M	non-Hispanic White	27	Some college	16	Non-menthol	Not thinking about quitting
20	F	non-Hispanic White	26	Bachelors degree	14	Non-menthol	Not thinking about quitting

ASI: age of smoking initiation

**Table 3:** Key themes generated by research objectives with sample quotes

*Category and descriptions*
**Objective 1: Explore adult daily cigarette smokers’ experiences using e-cigarettes in the context of smoke-free places/situations**
**Public places/situations of use**
“The first time [e-cigarette use]…I was walking down the street when I left here.” (No.6, Male, 58 years)
“I’ll just leave it [e-cigarette] on my register or whatever like that and I’ll just grab like a draw or two off of it.” (No.17, Male, 32 years)
“It’s [e-cigarette] not dissatisfying to people that be around me, like at work. I told them what it was and everything and they said we don’t smell anything.” (No.15, Male, 56 years)
“I got on the bus. I didn’t have no cigarette to put out. I just kept it [e-cigarette] in my mouth while I scanned my card. (No.7, Male, 24 years)
“We sat on the train and we was waiting for it to pull off. I pulled out my e-cigarette right there and I was puffing.” (No.1, Female, 55 years)
**Private places/situations of use**
“I used them [e-cigarettes] mostly in my bedroom.” (No.14, Female, 29 years)
“I smoked [e-cigarette] in the car. I smoke it in the house.” (No.19, Male, 27 years)
“I think because I’ve been smoking the e-cigarettes only inside and I don’t typically smoke inside anywhere, and I don’t know if it’s [e-cigarette use] allowed or whatever, I mean, all these other places. I know that I’m allowed to do that [e-cigarette use] at my house.” (No.12, Female, 37 years)
**Objective 2: Describe adult daily cigarette smokers’ perceptions of bystanders’ reactions to them using e-cigarettes in smoke-free places/situations**
**Favorable**
“I feel like everybody wanted to smoke what I was smoking. Everybody, they seen me with it [e-cigarette] and they wanted to have it. I felt like I was a trendsetter or something.” (No.18, Male, 18 years)
**Unfavorable**
“I just walked on the bus. I took my seat. He didn’t say anything. The bus driver didn’t say nothing. So, I did about three or four puffs and then…they all did look at me like I was losing my mind, but I didn’t say anything. I just stopped dragging on it…put it back in its case…it’s a non-smoking bus.”(No.7, Male, 24 years)
**Impartial**
“I think because they’ve become more mainstream no one really reacted. Certainly, they didn’t say anything to me about using them.” (No.19, Male, 27 years)

### Themes

The results that follow are organized in relation to the study’s two research objectives. These include: 1) Explore adult daily cigarette smokers’ experiences using e-cigarettes in the context of smoke-free places; and 2) Describe adult daily cigarette smokers’ perceptions of bystanders’ reactions to them using e-cigarettes in smoke-free places/situations. We expand and discuss these themes in the sections below.

### Places of e-cigarette use

Throughout the narratives, participants reported using e-cigarettes in various public and private places, such as their homes/indoors, public transportation, private vehicles, workplaces, and bars/restaurants. One participant reported that he first used an e-cigarette after his second interview: *‘The first time [e-cigarette use]…I was walking down the street when I left here.’ (No.6, Male, 58 years)*. Another participant reported in her interviews that she was more likely to use e-cigarettes at home. When asked about where she used e-cigarettes, she replied: *‘I used them [e-cigarettes] mostly in my bedroom.’ (No.14, Female, 29 years)*. In addition to using e-cigarettes inside his house, a participant reported in his interviews that he used e-cigarettes in his personal vehicle: *‘I smoked [e-cigarette] in the car. I smoke it in the house. It was okay too because it doesn’t have that smell or nothing like…it’s just smoke.’ (No.19, Male, 27 years)*. The use of e-cigarettes inside his house and vehicle suggests that indoor places are more conducive to e-cigarette use than cigarette smoking because of the lack of tobacco smell.

Some participants described how they integrated e-cigarettes into their daily work routine. For example, one participant said: ‘on my breaks, it’s easier to do it [use e-cigarettes].’ *(No.3, Female, 42 years)*. Another participant, who reported being employed at a restaurant, stated: *‘I’ve just been trying to do it [use e-cigarettes] where I like.’ (No.17, Male, 32 years)*. He explained:

‘I’ll just leave it [e-cigarette] on my register or whatever like that and I’ll just grab like a draw or two off of it. Then you know, it might be another hour or two before I even get back to it. I might have like another draw or two off of it, versus having to go take like a five or 10-minute break, have somebody cover my register, then I’ll go smoke a cigarette or two.’ (No.17, Male, 32 years).

For these participants, e-cigarette use at work was perceived to be convenient and beneficial, as an alternative to routine smoke breaks, and in turn, an uninterrupted work shift. Similarly, another interviewee stated: *‘It’s [e-cigarette] not dissatisfying to people that be around me, like at work. I told them what it was and everything and they said we don’t smell anything.’ (No.15, Male, 56 years)*. His viewpoint indicates that e-cigarette use at work is socially acceptable, because unlike cigarettes, e-cigarettes do not produce a smell that is offensive to his co-workers.

While some participants used e-cigarettes at work, other participants reported in their interviews that they restricted e-cigarette use to inside their house during the trial period. For example, one participant expressed uncertainty about e-cigarette use in places/situations other than her house: *‘I think because I’ve been smoking the e-cigarettes only inside and I don’t typically smoke inside anywhere, and I don’t know if it’s [e-cigarette use] allowed or whatever, I mean, all these other places. I know that I’m allowed to do that [e-cigarette use] at my house.’ (No.12, Female, 37 years)*. According to this participant, e-cigarette use inside her home is acceptable and free of unintended consequences. In the exchange given below, a participant conveyed to the interviewer that inside her house was more conducive to e-cigarette use than cigarette smoking, and how e-cigarette use in outside places would feel strange:

‘Yeah, I didn’t have to go out so that was kind of cool. I can’t really ever imagine using it [e-cigarette] outside. That would be weird to me.’ (No.14, Female, 29 years) ‘Why?’ (Interviewer)

‘Because why would you go outside? I guess…if I’m already outside. I don’t know, it just feels like…I like regular cigarettes more, so that’s what I’m going to do if I’m outside.’ (No.14, Female, 29 years).

Her remarks suggest that bans on smoking in enclosed places have normalized cigarette smoking, not e-cigarette use, as typical outside behavior. She also states that she likes cigarettes more than e-cigarettes, and that she’s willing to go outside to smoke cigarettes, suggesting how a smoker might weigh the pros and cons of switching from cigarettes to e-cigarettes. Similarly, when asked if she used e-cigarettes outside, another participant reported that she only used e-cigarettes outside as a cigarette substitute when she ran out of cigarettes.

‘I remember you said last time you were using them in the house.’ (Interviewer)

‘Yes.’ (No.2, Female, 28 years)

‘Okay. Did you use it [e-cigarette] at all outside?’ (Interviewer)

‘Not really. Like if I didn’t have any more cigarettes I just smoked them [e-cigarettes]’ (No.2, Female, 28 years)

### Perceived reactions from bystanders

In their second and third interviews, participants described trying to test and establish the social and spatial boundaries of e-cigarette use while in smoke-free places and recounted divergent experiences of their perceptions of how bystanders reacted to them using e-cigarettes in smoke-free environments. Perceived bystanders’ reactions were described as impartial, unfavorable, or favorable. According to one participant, the widespread use of e-cigarettes in public places is the reason bystanders did not react negatively or positively to his e-cigarette use: *‘I think because they’ve become more mainstream no one really reacted. Certainly, they didn’t say anything to me about using them.’ (No.19, Male, 27 years).* Similarly, a participant who reported using e-cigarettes in bars to circumvent smoke-free polices, described having no reactions from bystanders. He said: *‘You see a lot of them [e-cigarettes] out at the bar actually. You kind of get away with it inside.’ (No.11, Male, 29 years).*

However, another interviewee modified his e-cigarette use in response to negative reactions. He reflected on bystanders’ reactions to him using e-cigarettes inside the bus:

‘I got on the bus. I didn’t have no cigarette to put out. I just kept it [e-cigarette] in my mouth while I scanned my card. I just walked on the bus. I took my seat. He didn’t say anything. The bus driver didn’t say nothing. So, I did about three or four puffs and then…they all did look at me like I was losing my mind, but I didn’t say anything. I just stopped dragging on it…put it back in its case…it’s a non-smoking bus.’(No.7, Male, 24 years).

When asked if bystanders told him it’s ‘a non-smoking bus’, he remarked: *‘No, nobody said nothing.’ (No.7, Male, 24 years).* This suggests that the negative facial expressions from other passengers signaled a social cue that using e-cigarettes, like smoking cigarettes, is unacceptable and prohibited inside the bus. Another interviewee reported that she was very aware of her e-cigarette use inside the train. Recalling how her friend initially thought she was about to smoke a cigarette, she explained that she anticipated bystanders’ possible negative reaction to her e-cigarette use:

‘We sat on the train and we was waiting for it to pull off. I pulled out my e-cigarette right there and I was puffing, and then he said, ‘you know you can’t’ …then he said, ‘oh shoot, that’s them fake outs’ [laughs]. People would look for a second, but then I guess maybe they realized it wasn’t, you know, a real cigarette after they would look. But, a couple of people looked at me and you know, they turned like they wasn’t trying to look, but they looked, and I would see their reaction. That’s what I was going for though. I wanted to see people’s reaction. And, I’m going to try that again, you know, somewhere like at a restaurant or something, I’m going to try it again. I want to see their reactions.’ (No.1, Female, 55 years).

She went on to describe how being able to satisfy her desire to smoke a cigarette at that moment, motivated her e-cigarette use inside the train:

*‘It felt good to be able to smoke [e-cigarette] because we had like maybe 10-15 minutes before the train pulled off…so I’m like, okay we can’t go back off to smoke a cigarette or we got to pay again, and I wanted something to puff on so I pulled it [e-cigarette] out and I just sat there and puffed on it. And I felt good I was able to do that.’(No.1, Female, 55 years)*. Another participant also reported e-cigarette use inside the train. Describing himself as a ‘militant smoker’ in his first interview, he mentioned that an appealing commercial motivated him to try e-cigarettes and counter the cigarette smoking stigma. He explained:

‘I was kind of a militant smoker. It was like, you know, you’re not going to push me out of society because I smoke…it just happens to be a habit that I have. I understand that it’s uncomfortable for some people and distasteful for some people, but it’s my right to smoke. And, that’s what the sort of gist of the commercial was. You know, regain your independence…because as I said, I do enjoy the ingesting of the smoke. If there was some way that it [e-cigarette] doesn’t bother other people…if it [e-cigarette] doesn’t encroach in sort of their personal space, I think that’s like a brilliant invention, if it [e-cigarette] works.’ (No.5, Male, 61 years).

After initiating e-cigarettes, he reported in his second interview that he tested the social and spatial boundaries of e-cigarette use inside the train but not inside other smoke-free places that he frequents, like church and restaurants:

‘I was with a friend who was fearful that we would get arrested [laughs]. There were people around and there was no reaction. I was thinking I didn’t feel I had the guts enough to really kind of like, do it [use e-cigarette] in church or [laughs]…do it in restaurants, to really sort of test that, but public transportation was enough. I think even though I talked about being a kind of militant smoker, I’m a coward I suppose…when it comes to drawing a line in the sand… at least in terms of smoking, you know. I exert my rights, but perhaps if I got more comfortable with using it [e-cigarette] you know, I would definitely give it [e-cigarette use in church or restaurants] a shot.’ (No.5, Male, 61 years).

Although bystanders did not react negatively or positively to his e-cigarette use inside the train, he described a complex decisional balance influenced by fear, perceived rights as a smoker, and a lack of comfort using e-cigarettes that prevented him from ‘testing’ other smoke-free places.

However, one participant described himself as a ‘trendsetter’, and articulated positive reactions from bystanders and social acceptance: *‘I feel like everybody wanted to smoke what I was smoking. Everybody, they seen me with it [e-cigarette] and they wanted to have it. I felt like I was a trendsetter or something.’ (No.18, Male, 18 years).* Additionally, in his third interview, he reported that he used e-cigarettes inside a restaurant and recalled the initial reaction from bystanders, including the security guard:

‘I’m smoking inside…they thought it [e-cigarette] was a regular cigarette. I puffed. Like, I ate, and then I puffed it. The security guard came over, he was like, ‘You know you can’t smoke in here,’ all this other stuff…’You can get arrested for that’. I was like ‘This is the e-cigarette’. He said, ‘Alright,’ and walked off.’(No.18, Male, 18 years).

His remark that ‘this is the e-cigarette’, and the security guard’s consensus, suggests that e-cigarette use inside the restaurant is permissible because e-cigarettes are not cigarettes.

## DISCUSSION

This study is an initial attempt to investigate where, why and how adult daily smokers are trying to test and establish the social and spatial boundaries of e-cigarette use. The adult daily smokers in this study: 1) reported e-cigarette use in the context of indoor smoke-free policies (e.g. public transportation, restaurant, workplace) and inside their house; 2) described shoehorning themselves back into certain exclusionary spaces; 3) described uncertainty in relation to where they could use e-cigarettes; and 4) perceived that favorable, unfavorable, and impartial reactions from bystanders either facilitated or impeded their e-cigarette use, suggesting social approval/disapproval. These findings are a major step towards understanding the contexts of e-cigarette use from the perspective of smokers initiating e-cigarettes. As mentioned previously, during the time these interviews were conducted, the public smoking ban in Washington, DC had not yet been extended to e-cigarettes. Further research is needed to investigate how smokers initiating e-cigarettes are aware of and interact with public smoking bans that have been extended to e-cigarettes, and how these bans affect cigarette smoking and e-cigarette use.

Several participants described how e-cigarettes operated as props that signaled to bystanders that they were attempting to test the social and spatial boundaries of e-cigarette use. While some participants associated favorable reactions with social approval, others associated unfavorable reactions with social disapproval, and described how unfavorable reactions impeded e-cigarette use in smoke-free places/situations. Further, participants who perceived impartial reactions from bystanders described e-cigarette use to be common inside places where smoking is prohibited in Washington, DC, such as bars, where e-cigarettes were used to circumvent smoke-free policies. While state and localities are considering whether to permit or prohibit e-cigarettes in smoke-free public places, our findings suggest that it is important to consider whether smokers initiating e-cigarettes view smoke-free policies as facilitating or impeding e-cigarette use. Some participants reported that smoke-free policies have prompted smokers to switch to using e-cigarettes in bars, without negative reactions or judgment. Other participants primarily limited e-cigarette use to inside their house, a space exclusive to them, making it possible to use the device unaccompanied by negative consequences. These findings suggest that smokers do not know where they can use e-cigarettes, which could affect those switching from cigarettes to e-cigarettes.

There have been studies^3,11,22^ suggesting that e-cigarettes are marketed as devices that can be used anywhere. Our current findings extend earlier studies by not only identifying where smokers use e-cigarettes, but also examining why they do or do not decide to use e-cigarettes in smoke-free places, and how they perceive bystanders’ reactions. The narratives in this study attest that approaches to using e-cigarettes are part of a complex decisional balance influenced by both individual and situational factors that range from testing the social and spatial boundaries reserved for non-smokers, to restricting e-cigarette use to inside the home. Moreover, participants viewed e-cigarette use indoors and outdoors as holding different meanings. While some participants discussed how they used e-cigarettes indoors (e.g. while eating at a restaurant) and outdoors (e.g. walking down the street), other participants described how outside/outdoor spaces were only used to smoke cigarettes, suggesting that smoke-free policies influence where smokers use e-cigarettes and that a smoker might weigh the pros and cons of switching from cigarettes to e-cigarettes. For example, some participants explicitly stated a preference for cigarettes and were willing to go outside to use them, but not e-cigarettes. This suggests that if smokers perceive that e-cigarettes cannot outcompete traditional cigarettes on nicotine delivery, taste, and ‘whole body’ satisfaction, then they may not switch to e-cigarettes^23^, and they may not comply with indoor smoke-free policies that prohibit e-cigarette use.

### Strengths and limitations

By employing repeated one-on-one interviews with adult daily cigarette smokers transitioning to e-cigarettes, our findings fill an existing gap in the literature and offer new explanations of why, where, and how e-cigarette use in smoke-free places/situations occurs. While the present study is based on a small and non-random sample that is justifiable for qualitative research^24^, an important caveat is the limited generalizability of the study’s findings beyond its 20 interviewees. The study does, however, offer new insights and raise important questions for further investigation. Providing adult smokers e-cigarettes and asking them to report back on their use via repeated individual interviews provides a novel way to collect qualitative data. Studies of the differences in reporting between naïve and established e-cigarette users are needed. Comparisons with adult daily e-cigarette-only users could also extend understanding of how users incorporate these devices into their daily lives. Further, comparisons to dual cigarette and e-cigarette users in rural areas could validate or invalidate the influence of the urban-suburban environment.

## CONCLUSIONS

This study builds upon existing qualitative research, as we identified several different experiences of adult smokers with e-cigarettes in smoke-free places/situations. The findings of this study provide us with a deeper understanding of environmental and place experiences and meanings that facilitate or impede e-cigarette use in smoke-free places/situations. Given the rapidly changing e-cigarette landscape^25^, investigating contexts specific to e-cigarette use among adult daily smokers may propel research and policy developed to evaluate the potential public health risks and benefits of e-cigarettes.
